# Thioridazine enhances sensitivity to carboplatin in human head and neck cancer cells through downregulation of c-FLIP and Mcl-1 expression

**DOI:** 10.1038/cddis.2017.8

**Published:** 2017-02-09

**Authors:** Seung Un Seo, Hyuk Ki Cho, Kyoung-jin Min, Seon Min Woo, Shin Kim, Jong-Wook Park, Sang Hyun Kim, Yung Hyun Choi, Young Sam Keum, Jin Won Hyun, Hyun Ho Park, Sang-Han Lee, Dong Eun Kim, Taeg Kyu Kwon

**Affiliations:** 1 Department of Immunology, School of Medicine, Keimyung University, 2800 Dalgubeoldaero, Dalseo-Gu, Daegu 704-701, South Korea; 2 Department of Otolaryngology, School of Medicine, Keimyung University, 2800 Dalgubeoldaero, Dalseo-Gu, Daegu 704-701, South Korea; 3 Deaprtment of Pharmacology, School of Medicine, Kyungpook National University, Daegu, South Korea; 4 Department of Biochemistry, College of Oriental Medicine, Dong-Eui University, Busan, South Korea; 5 Department of Biochemistry, College of Pharmacy, Dongguk University, Goyang 10326, South Korea; 6 School of Medicine, Jeju National University, Jeju 63243, South Korea; 7 School of Biotechnology and Graduate School of Biochemistry at Yeungnam University, Gyeongsan 38541, South Korea; 8 School of Food Science & Biotechnology, Kyungpook National University, Daegu 41566, Republic of Korea

## Abstract

Carboplatin is a less toxic analog of cisplatin, but carboplatin also has side effects, including bone marrow suppression. Therefore, to improve the capacity of the anticancer activity of carboplatin, we investigated whether combined treatment with carboplatin and thioridazine, which has antipsychotic and anticancer activities, has a synergistic effect on apoptosis. Combined treatment with carboplatin and thioridazine markedly induced caspase-mediated apoptosis in head and neck squamous cell carcinoma (AMC-HN4) cells. Combined treatment with carboplatin and thioridazine induced downregulation of Mcl-1 and c-FLIP expression. Ectopic expression of Mcl-1 and c-FLIP inhibited carboplatin plus thioridazine-induced apoptosis. We found that augmentation of proteasome activity had a critical role in downregulation of Mcl-1 and c-FLIP expression at the post-translational level in carboplatin plus thioridazine-treated cells. Furthermore, carboplatin plus thioridazine induced upregulation of the expression of proteasome subunit alpha 5 (PSMA5) through mitochondrial reactive oxygen species (ROS)-dependent nuclear factor E2-related factor 2 (Nrf2) activation. In addition, combined treatment with carboplatin and thioridazine markedly induced apoptosis in human breast carcinoma (MDA-MB231) and glioma (U87MG) cells, but not in human normal mesangial cells and normal human umbilical vein cells (EA.hy926). Collectively, our study demonstrates that combined treatment with carboplatin and thioridazine induces apoptosis through proteasomal degradation of Mcl-1 and c-FLIP by upregulation of Nrf2-dependent PSMA5 expression.

Carboplatin is an analog of cisplatin that binds to DNA and then inhibits replication and transcription, resulting in cell death.^1^ Carboplatin has less gastrointestinal toxicity, nephrotoxicity, and neurotoxicity compared with cisplatin.^2^ It is an effective chemotherapeutic drug in head and neck cancer,^3^ endometrial cancer,^4^ and small cell lung cancer.^5^ However, the anticancer effect of carboplatin is inhibited by intrinsic or acquired drug resistance. Several molecular mechanisms of carboplatin resistance have been discovered, including (1) decreased tumor blood flow and drug uptake, (2) increased drug efflux and detoxification, (3) increased DNA repair and tolerance of DNA damage, (4) downregulation of pro-apoptotic proteins and upregulation of antiapoptotic proteins, and (5) altered cell signaling pathways.^6^ Combination therapy with other anticancer drugs could improve the anticancer effect and reduce the drug resistance of carboplatin.

Thioridazine (10-[2-(1-methyl-2-piperidyl) ethyl]-2-(methylthio) phenothiazine) is an antipsychotic^7, 8^ and anti-microbial drug.^9, 10^ In addition, thioridazine also has anticancer activity through induction of apoptosis in multiple cancer cells^11, 12, 13, 14, 15, 16^ and inhibition of angiogenesis^17, 18^ and metastasis.^19, 20^ Furthermore, thioridazine overcomes drug resistance through inhibition of P-glycoprotein.^21, 22^ As high concentrations of thioridazine cause side effects, such as dysrhythmia and sudden death,^23, 24^ strategies against combined treatment with low concentrations of thioridazine plus anticancer drugs have been investigated. For example, combined treatment with thioridazine and TRAIL induced apoptosis in human renal carcinoma Caki cells by downregulating antiapoptotic proteins.^25^ In addition, treatment with thioridazine plus doxorubicin using nanoparticles inhibited growth in breast cancer cells.^26^

Degradation of proteins is mainly regulated by the ubiquitin-proteasome pathway (UPP). The 26S proteasome is a multi-protein complex and is composed of the 20 S catalytic proteasome and 19S regulatory subunits. The increase of proteasome activity is related to upregulation of the expression of the proteasome subunit.^27^ *De novo* synthesis of the proteasome subunit is tightly regulated by nuclear factor E2-related factor 2 (Nrf2).^28^ In the absence of stimuli, Keap-1 retains Nrf2 in the cytoplasm, resulting in inhibition of transcriptional activity. However, oxidative stress modifies the cysteine residue of Keap-1, and then, Nrf2 is released.^29^ The translocation of Nrf2 from the cytosol to the nucleus induces gene expression by binding to the antioxidant response element (ARE).^29^

In our study, we investigated whether combined treatment with carboplatin and thioridazine induces apoptosis and examined the molecular mechanisms of apoptosis in head and neck squamous cell carcinoma (AMC-HN4) cells.

## Results

### Combined treatment with carboplatin and thioridazine induces apoptosis

**Figure 1:**
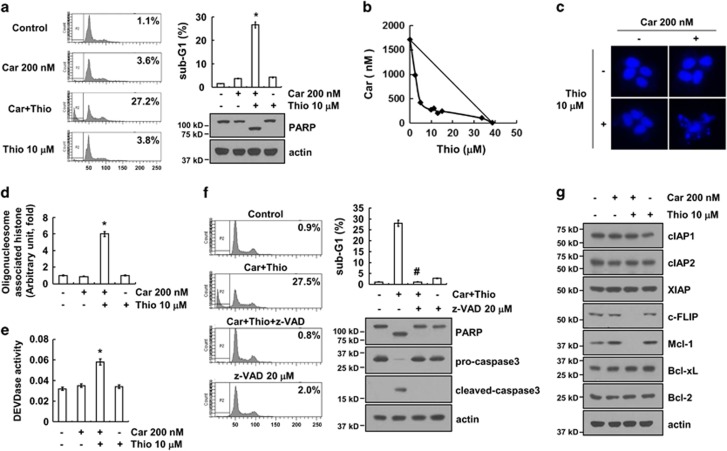
Combined treatment with carboplatin and thioridazine induces apoptosis in human head and neck cancer (AMC-HN4) cells. (**a**) AMC-HN4 cells were treated with 200 nM carboplatin (Car) in the presence or absence of 10 *μ*M thioridazine (Thio) for 24 h. The sub-G1 fraction was measured by flow cytometry as an indicator of the level of apoptosis. The protein expression levels of PARP and actin were determined by western blot. The level of actin was used as a loading control. (**b**) Isoboles were obtained by plotting the combined concentrations of each drug required to produce 50% cell death. The straight line connecting the IC_50_ values obtained for the two agents, when applied alone, corresponded to the addition of their independent effects. The values below this line indicate synergy, whereas the values above this line indicate antagonism. (**c**–**d**) AMC-HN4 cells were treated with 200 nM carboplatin in the presence or absence of 10 *μ*M thioridazine for 24 h. Condensation and fragmentation of the nuclei were detected by 4′,6′-diamidino-2-phenylindole staining (**c**). The cytoplasmic histone-associated DNA fragments were determined by a DNA fragmentation detection kit (**d**). Caspase activities were determined with colorimetric assays using caspase-3 (DEVDase) assay kits (**e**). (**f**) AMC-HN4 cells were treated with 200 nM carboplatin plus 10 *μ*M thioridazine for 24 h in the presence or absence of 20 *μ*M z-VAD-fmk (z-VAD). The sub-G1 fraction was measured by flow cytometry. The protein expression levels of PARP, pro-caspase-3, cleaved caspase-3, and actin were determined by western blotting. The level of actin was used as a loading control. (**g**) AMC-HN4 cells were treated with 200 nM carboplatin in the presence or absence of 10 *μ*M thioridazine for 24 h. The protein expression levels of cIAP1, cIAP2, XIAP, c-FLIP, Mcl-1, Bcl-xL, Bcl-2, and actin were determined by western blotting. The level of actin was used as a loading control. The values in **a**, **d**, **e**, and **f** represent the mean±S.D. from three independent samples. *P<0.01 compared with the control. ^#^*P*<0.01 compared with the combined treatment with carboplatin and thioridazine

To determine whether there is an apoptotic effect between carboplatin and thioridazine, AMC-HN4 cells were treated with carboplatin in the absence or presence of thioridazine. Carboplatin alone and thioridazine alone had no effect on apoptosis, but combined treatment with carboplatin and thioridazine induced apoptosis and PARP cleavage, which is a substrate of caspase-3 (Figure 1a). Next, we investigated whether combined treatment with carboplatin and thioridazine had synergistic effects. Isobologram analysis suggested that carboplatin plus thioridazine had synergistic effects (Figure 1b). Furthermore, carboplatin plus thioridazine increased chromatin damage in the nucleus (Figure 1c) and cytoplasmic histone-associated DNA fragments (Figure 1d). In addition, we examined whether caspase activation is involved in apoptosis in carboplatin plus thioridazine-treated cells. Combined treatment markedly increases caspase-3 activation (Figure 1e), and a pan-caspase inhibitor (z-VAD) blocked carboplatin and thioridazine-induced apoptosis as well as PARP cleavage (Figure 1f). To further determine the molecular mechanisms underlying carboplatin plus thioridazine-induced apoptosis, we examined the modulation of the expression of apoptosis-related proteins. Both c-FLIP and Mcl-1 expression were downregulated (Figure 1g). These results indicated that combined treatment with carboplatin and thioridazine induces caspase-mediated apoptosis and downregulated expression of c-FLIP and Mcl-1.

### Ectopic expression of c-FLIP and Mcl-1 overcomes apoptosis in carboplatin plus thioridazine-treated cells

**Figure 2:**
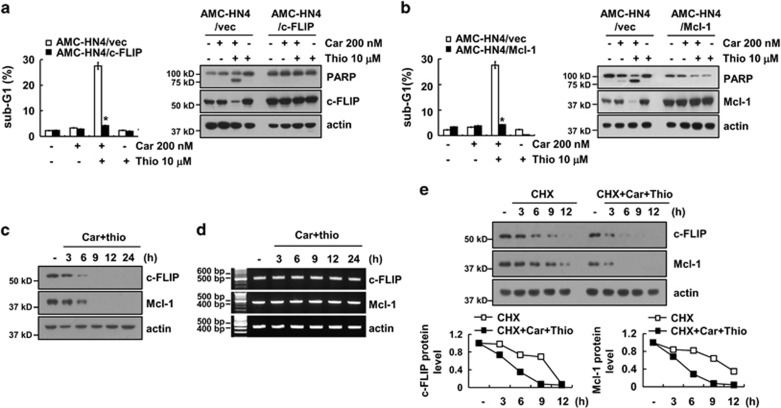
Downregulation of c-FLIP and Mcl-1 expression by carboplatin plus thioridazine contributes to apoptosis. (**a** and **b**) AMC-HN4 cells were transiently transfected with pcDNA 3.1-c-FLIP (**a**) or pFLAG-CMV-4/Mcl-1 (**b**). Twenty-four hours after transfection, cells were treated with 200 nM carboplatin in the presence or absence of 10 *μ*M thioridazine for 24 h. The sub-G1 fraction was measured by flow cytometry. The protein expression levels of PARP, c-FLIP, Mcl-1, and actin were determined by western blotting. The level of actin was used as a loading control. (**c** and **d**) AMC-HN4 cells were treated with 200 nM carboplatin plus 10 *μ*M thioridazine for the indicated time periods. The protein and mRNA expression levels of c-FLIP, Mcl-1, and actin were determined by western blotting and RT-PCR, respectively. The level of actin was used as a loading control. (**e**) AMC-HN4 cells were treated with or without 200 nM carboplatin plus 10 *μ*M thioridazine in the presence of 20 *μ*g/ml cyclohexamide (CHX) for the indicated time periods. The protein expression levels of c-FLIP, Mcl-1, and actin were determined by western blotting. The level of actin was used as a loading control. The band intensity of the c-FLIP and Mcl-1 protein was measured using ImageJ (public domain JAVA image-processing program ImageJ (http://rsb.info.nih.gov/ij). The values in **a** and **b** represent the mean±S.D. from three independent samples. *P<0.01 compared with carboplatin plus thioridazine-treated AMC-HN4/Vec

To evaluate the functional importance of the c-FLIP and Mcl-1 proteins in carboplatin plus thioridazine-induced apoptosis, we used c-FLIP- and Mcl-1-overexpressing cells. Overexpression of c-FLIP or Mcl-1 markedly inhibits carboplatin plus thioridazine-induced apoptosis and PARP cleavage (Figure 2a and b). Combined treatment with carboplatin and thioridazine gradually decreased c-FLIP and Mcl-1 expression over 6 h (Figure 2c), but the mRNA expression of both was not changed (Figure 2d). Therefore, we examined whether carboplatin plus thioridazine modulates the protein stability of c-FLIP and Mcl-1. Cells were treated with cycloheximide (CHX), an inhibitor of *de novo* protein synthesis, in the presence or absence of carboplatin plus thioridazine. As shown in Figure 2e, CHX in the presence of carboplatin plus thioridazine rapidly reduced expression of c-FLIP and Mcl-1 compared with CHX alone. These data suggested that the combined treatment of carboplatin and thioridazine induces downregulation of c-FLIP and Mcl-1 expression at the post-translational level and that downregulation of c-FLIP and Mcl-1 expression has a critical role in carboplatin plus thioridazine-induced cell death.

### Upregulation of PSMA5 expression is associated with carboplatin plus thioridazine-induced apoptosis

**Figure 3:**
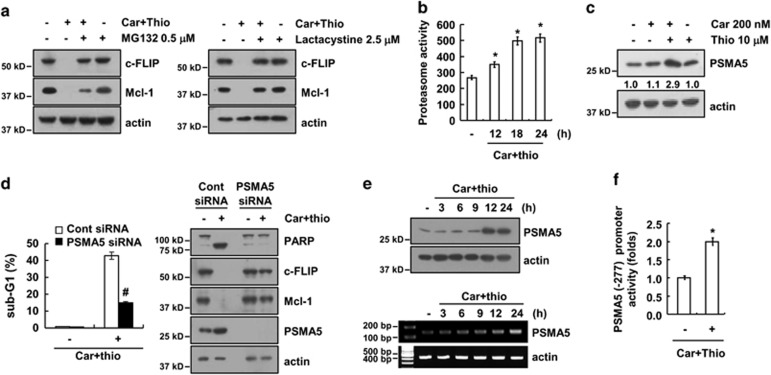
Combined treatment with carboplatin and thioridazine upregulated the expression of PSMA5. (**a**) AMC-HN4 cells were pretreated with 0.5 *μ*M MG132 and 2.5 *μ*M lactacystin for 30 min and were then combined with 200 nM carboplatin plus 10 *μ*M thioridazine for 24 h. The protein expression levels of c-FLIP, Mcl-1, and actin were determined by western blotting. The level of actin was used as a loading control. (**b**) AMC-HN4 cells were treated with 200 nM carboplatin plus 10 *μ*M thioridazine for the indicated time periods. After treatment, the cells were lysed, and the proteasome activity was measured as described in the Materials and Methods section. (**c**) AMC-HN4 cells were treated with 200 nM carboplatin in the presence or absence of 10 *μ*M thioridazine for 24 h. The protein expression levels of PSMA5 and actin were determined by western blotting. The level of actin was used as a loading control. The band intensity of the PSMA5 protein was measured using ImageJ (public domain JAVA image-processing program ImageJ (http://rsb.info.nih.gov/ij). (**d**) AMC-HN4 cells were transiently transfected with a control siRNA or PSMA5 siRNA. Twenty-four hours after transfection, the cells were treated with 200 nM carboplatin plus 10 *μ*M thioridazine for 24 h. The sub-G1 fraction was measured by flow cytometry as an indicator of the level of apoptosis. The protein expression levels of PARP, c-FLIP, Mcl-1, PSMA5, and actin were determined by western blotting. The level of actin was used as a loading control. (**e**) AMC-HN4 cells were treated with 200 nM carboplatin plus 10 *μ*M thioridazine for the indicated time periods. The protein and mRNA expression levels of PSMA5 and actin were determined by western blotting and RT-PCR, respectively. (**f**) AMC-HN4 cells were transiently transfected with a plasmid harboring the luciferase gene under the control of the PSMA5/-277 promoter. After transfection, the cells were treated with 200 nM carboplatin plus 10 *μ*M thioridazine for 24 h. The luciferase activity was analyzed. The values in**b**, **d**, and **f** represent the mean±S.D. from three independent samples. *P<0.01 compared with the control. ^#^*P*<0.01 compared with the carboplatin plus thioridazine-treated control siRNA

Downregulation of c-FLIP and Mcl-1 protein expression is mainly regulated by the activation of the Ubiquitin-proteasome pathway (UPP).^30, 31^ As shown in Figure 3a, proteasome inhibitors (MG132 and lactacystine) inhibited carboplatin plus thioridazine-mediated downregulation of c-FLIP and Mcl-1 protein expression, and proteasome activity was markedly increased by the combined treatment with carboplatin and thioridazine (Figure 3b). Therefore, we investigated whether carboplatin plus thioridazine modulates the expression of the key proteasome subunit, 20 S proteasome subunit alpha type 5 (PSMA5). PSMA5 expression is markedly upregulated by the carboplatin plus thioridazine treatment (Figure 3c). Next, we examined whether upregulation of PSMA5 expression has a critical role in carboplatin plus thioridazine-mediated apoptosis. As shown in Figure 3d, downregulation of PSMA5 by siRNA inhibited apoptosis and cleavage of PARP in carboplatin plus thioridazine-treated cells. In addition, the combined treatment-mediated downregulation of c-FLIP and Mcl-1 was recovered by knockdown of PSMA5 (Figure 3d). The combined treatment with carboplatin and thioridazine upregulated the expression of PSMA5 at the protein and mRNA level in a time-dependent manner (Figure 3e). The PSMA5 promoter activity was also increased with the carboplatin plus thioridazine treatment (Figure 3f). Therefore, our data indicate that upregulation of PSMA5 has a critical role in carboplatin plus thioridazine-mediated apoptosis by downregulating expression of c-FLIP and Mcl-1.

### The Nrf2/ARE signaling pathway is largely indispensable for carboplatin plus thioridazine-mediated upregulation of PSMA5 expression

**Figure 4:**
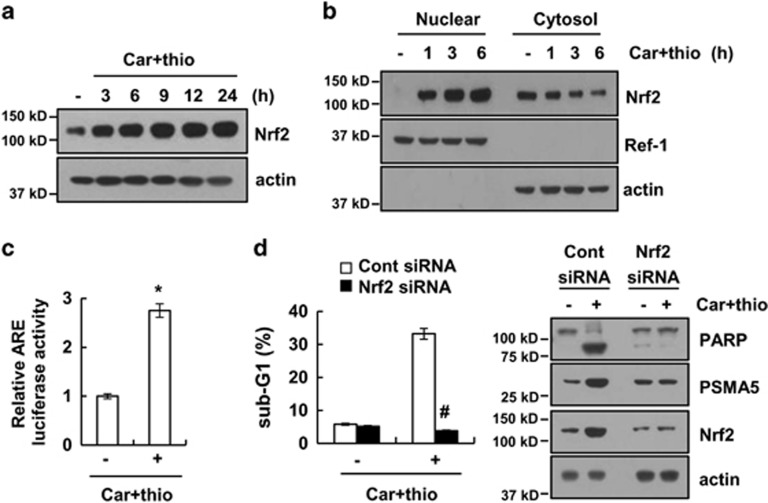
Combined treatment with carboplatin and thioridazine induced PSMA5 expression in an Nrf2-dependent manner. (**a**) AMC-HN4 cells were treated with 200 nM carboplatin plus 10 *μ*M thioridazine for the indicated time periods. The protein expression levels of Nrf2 and actin were determined by western blotting. The level of actin was used as a loading control. (**b**) AMC-HN4 cells were treated with 200 nM carboplatin plus 10 *μ*M thioridazine for the indicated time periods. After treatment, the nuclear extracts and cytosolic extracts were analyzed for Nrf2 and Ref-1 by western blotting as described in the Materials and Methods. Ref-1 was used as a marker for the nuclear fraction. (**c**) AMC-HN4 cells were transfected with an ARE-luciferase construct for 24 h, and the cells were treated with 200 nM carboplatin plus 10 *μ*M thioridazine for 24 h. After treatment, the cells were lysed and assayed for luciferase activity. (**d**) AMC-HN4 cells were transiently transfected with a control siRNA or Nrf2 siRNA. Twenty-four hours after transfection, the cells were treated with 200 nM carboplatin plus 10 *μ*M thioridazine for 24 h. The sub-G1 fraction was measured by flow cytometry as an indicator of the level of apoptosis. The protein expression levels of PARP, PSMA5, Nrf2, and actin were determined by western blotting. The values in **c** and **d** represent the mean±S.D. from three independent samples. *P<0.01 compared with control. ^#^*P*<0.01 compared with carboplatin plus thioridazine-treated control siRNA

Previous studies reported that the Nrf2/ARE signaling pathway is associated with the regulation of PSMA5 expression.^28^ Therefore, we investigated whether the Nrf2/ARE pathway is associated with PSMA5 expression in carboplatin plus thioridazine-treated cells. As shown in Figure 4a, carboplatin plus thioridazine induced upregulation of Nrf2 expression. Furthermore, nuclear translocation of Nrf2 is induced within 1 h and increased up to 6 h after carboplatin plus thioridazine treatment (Figure 4b). Combined treatment with carboplatin plus thioridazine induced the transcriptional activity of ARE (Figure 4c). To identify the importance of Nrf2/ARE signaling on carboplatin plus thioridazine-induced apoptosis, cells were treated with Nrf2 siRNA. Downregulation of Nrf2 expression by siRNA inhibited apoptotic cell death and upregulated PSMA5 expression (Figure 4d). Therefore, these data suggest that the combined treatment triggers upregulation of PSMA5 expression in an Nrf2/ARE signaling pathway-dependent manner.

### Generation of ROS is critically required for carboplatin plus thioridazine-induced apoptosis

**Figure 5:**
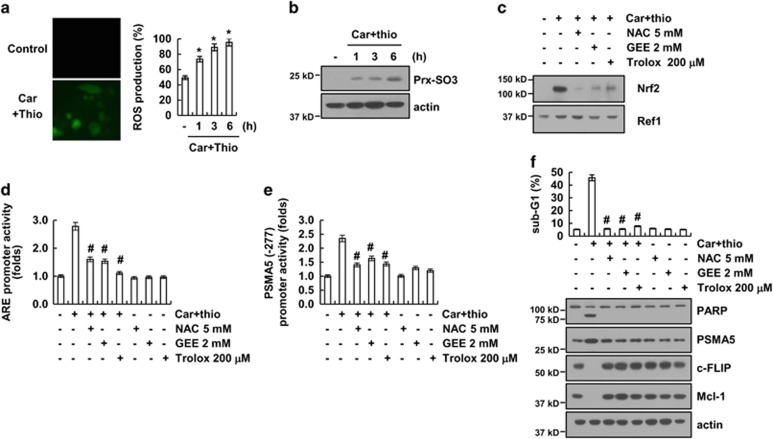
Reactive oxygen species has a critical role in carboplatin plus thioridazine-mediated PSMA5 expression. (**a**) AMC-HN4 cells were treated with 200 nM carboplatin plus 10 *μ*M thioridazine for 6 h (left panel) or the indicated time periods (right panel), and the cells were then loaded with the H_2_DCF-DA fluorescent dye. The H_2_DCF-DA fluorescence intensity was detected by a fluorescence microscope (left panel) and flow cytometry (right panel). (**b**) AMC-HN4 cells were treated with 200 nM carboplatin plus 10 *μ*M thioridazine for the indicated time periods. The protein expression levels of Prx-SO3 and actin were determined by western blotting. The level of actin was used as a loading control. (**c**) AMC-HN4 cells were pretreated with 5 mM NAC, 2 mM GEE, and 200 *μ*M trolox for 30 min and were then treated with 200 nM carboplatin plus 10 *μ*M thioridazine for 24 h. After treatment, the nuclear extracts were analyzed for Nrf2 and Ref-1 by western blotting as described in the Materials and Methods. Ref-1 was used as a marker of the nuclear fraction. (**d**) AMC-HN4 cells were transfected with an ARE-luciferase construct for 24 h. The cells were pretreated with 5 mM NAC, 2 mM GEE, and 200 *μ*M trolox for 30 min and were then treated with 200 nM carboplatin plus 10 *μ*M thioridazine for 24 h. After treatment, the cells were lysed and assayed for luciferase activity. (**e**) AMC-HN4 cells were transiently transfected with a plasmid harboring the luciferase gene under the control of the PSMA5/-277 promoter. After transfection, the cells were pretreated with 5 mM NAC, 2 mM GEE, and 200 *μ*M trolox for 30 min and were then treated with 200 nM carboplatin plus 10 *μ*M thioridazine for 24 h. The luciferase activity was analyzed. (**f**) AMC-HN4 cells were pretreated with 5 mM NAC, 2 mM GEE, and 200 *μ*M trolox for 30 min and were then treated with 200 nM carboplatin plus 10 *μ*M thioridazine for 24 h. The sub-G1 fraction was measured by flow cytometry. The protein expression levels of PARP, PSMA5, c-FLIP, Mcl-1, and actin were determined by western blotting. The level of actin was used as a loading control. The values in **a**, **d**, **e** and **f** represent the mean±S.D. from three independent samples. *P<0.01 compared with the control. ^#^*P*<0.01 compared with the carboplatin plus thioridazine

As the Nrf2/ARE signaling pathway is activated by reactive oxygen species (ROS) production,^32^ we investigated whether combined treatment with carboplatin and thioridazine induces ROS production. Combined treatment with carboplatin and thioridazine induced ROS production within 1 h (Figure 5a). In addition, we determined intracellular ROS by measuring the expression level of peroxiredoxin (Prx)-SO3. As shown in Figure 5b, peroxiredoxin (Prx)-SO3 was detected within 1 h in carboplatin plus thioridazine-treated cells. Next, we investigated whether ROS are involved in the activation of Nrf2/ARE signaling. We found that ROS scavengers *(N*-acetylcysteine (NAC), glutathione-ethyl-ester, and trolox) inhibited the nuclear translocation of Nrf2 as well as ARE transcriptional activity in carboplatin plus thioridazine-treated cells (Figure 5c and d). The carboplatin plus thioridazine-induced PSMA5 promoter activity was also reduced by ROS scavengers (Figure 5e). Furthermore, ROS scavengers inhibited apoptotic cell death, downregulated c-FLIP and Mcl-1 expression, and upregulated PSMA5 expression in carboplatin plus thioridazine-treated cells (Figure 5f). Therefore, our results suggest that ROS have a critical role in carboplatin plus thioridazine-induced apoptosis.

### Mitochondrial ROS have an important role in carboplatin plus thioridazine-induced apoptosis

**Figure 6:**
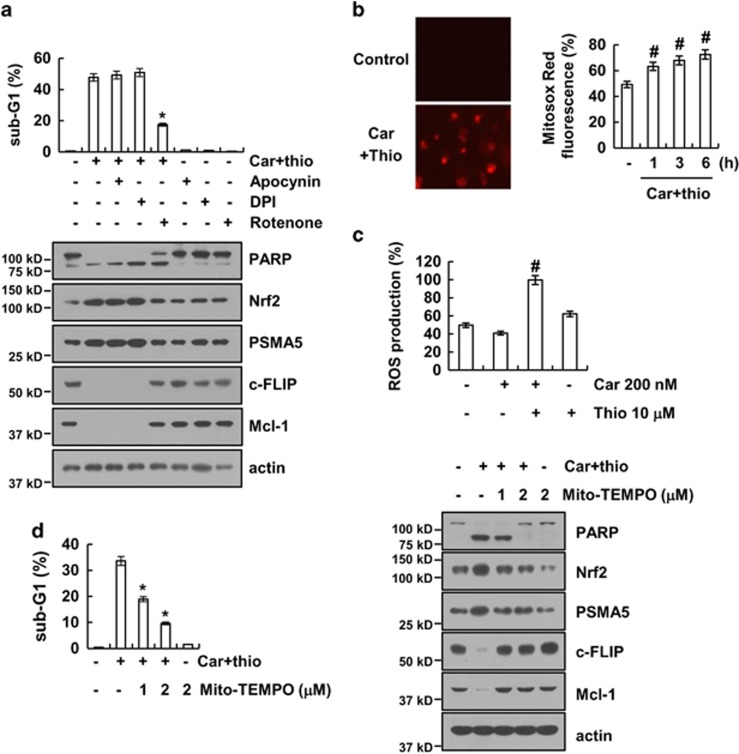
Mitochondrial reactive oxygen species are important for carboplatin plus thioridazine-induced apoptosis. (**a**) AMC-HN4 cells were pretreated with 100 *μ*M apocynin, 50 nM DPI, and 20 nM rotenone for 30 min followed by stimulation with 200 nM carboplatin plus 10 *μ*M thioridazine for 24 h. The sub-G1 fraction was measured by flow cytometry. The protein expression levels of PARP, Nrf2, PSMA5, c-FLIP, Mcl-1, and actin were determined by western blotting. The level of actin was used as a loading control. (**b**) AMC-HN4 cells were treated with 200 nM carboplatin plus 10 *μ*M thioridazine for 3 h (left panel) or the indicated time periods (right panel). The cells were then loaded with the Mitosox Red fluorescent dye. The Mitosox Red fluorescence intensity was detected by a fluorescence microscope (left panel) and flow cytometry (right panel). (**c**) AMC-HN4 cells were treated with 200 nM carboplatin in the presence or absence of 10 *μ*M thioridazine for 6 h. The cells were then loaded with the H_2_DCF-DA fluorescent dye. The H_2_DCF-DA fluorescence intensity was detected by flow cytometry. (**d**) AMC-HN4 cells were pretreated with the indicated concentrations of Mito-TEMPO and were then added with 200 nM carboplatin plus 10 *μ*M thioridazine for 24 h. The sub-G1 fraction was measured by flow cytometry. The protein expression levels of PARP, Nrf2, PSMA5, c-FLIP, Mcl-1, and actin were determined by western blotting. The level of actin was used as a loading control. The values in **a**, **b**,**c**, and **d** represent the mean±S.D. from three independent samples. *P<0.01 compared with the carboplatin plus thioridazine. ^#^*P*<0.01 compared with the control

As shown in Figure 5f, ROS have an important role in apoptosis induced by carboplatin plus thioridazine. Therefore, we investigated the source of ROS production induced by carboplatin plus thioridazine. ROS are mainly produced by NADPH oxidase and the mitochondrial electron transport chain.^33^ Cells were treated with NADPH oxidase inhibitors (diphenyleneiodonium (DPI) and apocynin) and a mitochondrial complex I inhibitor (rotenone).^34, 35^ Rotenone reduced carboplatin plus thioridazine-induced apoptotic cell death and cleavage of PARP, but DPI and apocynin did not (Figure 6a). In addition, rotenone reversed the upregulation of Nrf2 and PSMA5 expression and downregulated c-FLIP and Mcl-1 expression (Figure 6a). As shown in Figure 6b, combined treatment with carboplatin and thioridazine induced mitochondrial ROS within 1 h. Carboplatin alone or thioridazine alone did not significantly increase ROS production at the concentrations used in this study. However, the combined treatment markedly produced ROS (Figure 6c). To confirm the importance of mitochondrial ROS, we used a specific mitochondrial superoxide scavenger, Mito-TEMPO. Mito-TEMPO markedly reduced apoptosis and the cleavage of PARP in a dose-dependent manner (Figure 6d). Upregulation of Nrf2 and PSMA5 and downregulation of c-FLIP and Mcl-1 were also reversed by Mtio-TEMPO treatment in carboplatin and thioridazine-treated cells (Figure 6d). Therefore, our data suggest that mitochondrial ROS have a critical role in carboplatin and thioridazine-induced apoptosis.

### Combined treatment with carboplatin and thioridazine induces apoptosis in other cancer cells but not in normal cells

**Figure 7:**
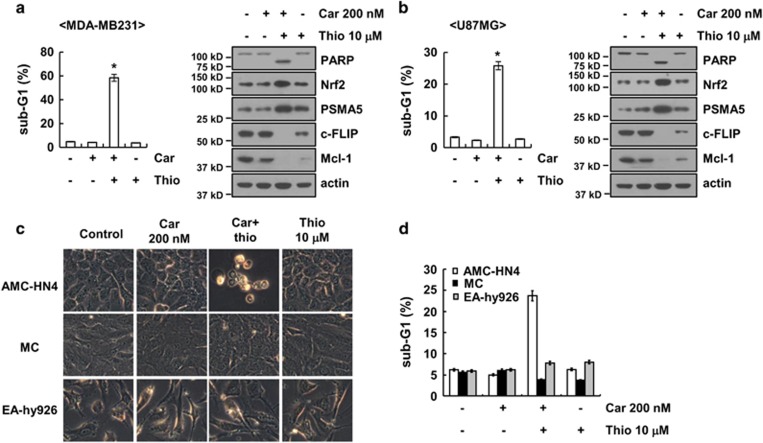
Effect of carboplatin plus thioridazine on apoptosis in other cancer cells and in normal cells. (**a** and **b**) Breast cancer (MDA-MB231) and glioma (U87MG) cells were treated with 200 nM carboplatin plus 10 *μ*M thioridazine for 24 h. The sub-G1 fraction was measured by flow cytometry. The protein expression levels of PARP, Nrf2, PSMA5, c-FLIP, Mcl-1, and actin were determined by western blotting. The level of actin was used as a loading control. (**c** and **d**) AMC-HN4 cells, mesangial cells (MC), and normal human umbilical vein cells (EA.hy926) were treated with 200 nM carboplatin plus 10 *μ*M thioridazine for 24 h. The cell morphology was examined using interference light microscopy (**c**). The sub-G1 fraction was measured by flow cytometry (**d**). The values in **a**, **b**, and **d** represent the mean±S.D. from three independent samples. *P<0.01 compared with the control

We next investigated the effect of carboplatin plus thioridazine on apoptosis in other cancer cells, including human breast carcinoma (MDA-MB231) and glioma (U87MG) cells. We found that combined treatment with carboplatin and thioridazine induced apoptotic cell death and cleavage of PARP in MDA-MB231 and U87MG cells (Figure 7a and b). Furthermore, carboplatin plus thioridazine induced upregulation of Nrf2 and PSMA5 expression and downregulation of c-FLIP and Mcl-1 expression (Figure 7a and b). By contrast, combined treatment with carboplatin and thioridazine did not induce morphological changes or apoptosis in normal human mesangial cells and normal human umbilical vein cells (EA.hy926) (Figure 7c and d).

## Discussion

In this study, we demonstrated that carboplatin plus thioridazine induces apoptosis in cancer cells, but not in normal cells. Combined treatment with carboplatin and thioridazine downregulated c-FLIP and Mcl-1 expression at the post-translational level in a proteasome-dependent manner. Along with the augmentation of proteasome activity, mitochondrial ROS activated the Nrf2/ARE signaling pathway and induced upregulation of PSMA5 expression in carboplatin plus thioridazine-treated cells.

Multiple mechanisms are involved in the anticancer activity of thioridazine. First, thioridazine inhibits PI3K/Akt signaling, which is important for cancer cell survival. Thioridazine inhibits cell viability and induces cell death through inhibition of the PI3K/Akt signaling pathway in ovarian cancer and in cervical and endometrial cancer cells.^11, 12^ In addition, thioridazine inhibits angiogenesis and tumor growth in ovarian cancer xenografts by inhibiting PI3K/Akt signaling.^17^ In our study, thioridazine also inhibited Akt phosphorylation (Supplementary Figure S1a). However, PI3K/Akt inhibitors (LY294002 and wortmanin) plus carboplatin did not induce apoptosis in head and neck cancer cells (Supplementary Figure S1b). Furthermore, both PI3K/Akt inhibitors had no effect on the downregulation of c-FLIP and Mcl-1 expression (Supplementary Figure S1b). Therefore, the anticancer effects of thioridazine are independent of the inhibition of the PI3K/Akt signaling pathways in head and neck cancer AMC-HN4 cells. Next, upregulation of ROS by thioridazine treatment also has a critical a role on the anticancer effect. Min *et al.*^25^ reported that thioridazine inhibited Mcl-1 and c-FLIP expression in a ROS-dependent manner, resulting in induction of apoptosis in TRAIL-treated human renal carcinoma Caki cells. However, thioridazine alone did not increase intracellular ROS levels (Figure 6c) and had no effect on the downregulation of c-FLIP and Mcl-1 expression (Figure 1g). Interestingly, a high concentration of carboplatin (>50 *μ*M) induced ROS production in cardiomyocytes,^36^ AMC-HN3 cells,^37^ and human laryngeal carcinoma cells.^38^ However, a low concentration of carboplatin (200 nM) did not increase ROS production in our experiment. In addition, Rodrigues *et al.*^39^ reported that thioridazine interacts with the membrane of mitochondria, acquiring antioxidant activity. By contrast, 10 *μ*M thioridazine induced ROS production in Caki cells. However, 10 *μ*M thioridazine had no effect on ROS production in AMC-HN4 cells. These effects of thioridazine on ROS production may result from the different cellular ROS statuses and cell contexts. Although thioridazine alone did not induce ROS production, combined treatment with carboplatin and thioridazine induced apoptosis by downregulating the expression of c-FLIP and Mcl-1 in a ROS-dependent manner. Finally, thioridazine inhibits the focal adhesion kinase (FAK) and Src signaling pathway. In ovarian cancer cells, thioridazine inhibits VEGF-induced angiogenesis by inhibiting FAK and Src phosphorylation.^18^ However, identification of the anticancer molecular mechanism of thioridazine requires further study.

When anticancer drugs induce apoptosis in cancer cells, the expression of apoptosis-related proteins is often regulated at the post-translational level.^25, 40^ Protein degradation is mainly regulated by the ubiquitin-proteasomal or lysosomal pathways. In this studies, combined treatment with carboplatin and thioridazine induced proteasome activity (Figure 3b), followed by downregulation of c-FLIP and Mcl-1 expression (Figure 3a). When proteins are degraded at the post-translational level, there are critical two steps: (1) augmentation of proteasome activity. In our study, combined treatment with carboplatin and thioridazine markedly increased proteasome activity. For the augmentation of proteasome activity, carboplatin plus thioridazine upregulated PSMA5 expression at the transcriptional level, which is a protein that has a 20 S proteasome catalytic core (Figure 3e). In previous studies, upregulation of the proteasome subunit was shown to be regulated by the Nrf2/ARE signaling pathways.^28, 41^ Combined treatment with carboplatin and thioridazine induced translocation of Nrf2 into the nucleus and then increased ARE transcriptional activity (Figure 4b and c). Downregulation of Nrf2 by siRNA inhibited upregulation of PSMA5 expression (Figure 4d). Interestingly, downregulation of PSMA5 expression by siRNA blocked augmentation of proteasome activity in carboplatin plus thioridazine-treated cells (Supplementary Figure S2). Therefore, upregulation of PSMA5 has a critical role on augmentation of proteasome activity. (2) upregulation of specific E3 ligase expression. As E3 ligase attaches ubiquitin to target proteins, protein degradation by the ubiquitin-proteasome system has high specificity. For example, four E3 ligases of Mcl-1 are known, including Mcl-1 ubiquitin ligase E3,^30^ beta transducin-containing protein (*β*-TrCP),^42^ F-box and WD repeat domain-containing 7,^43^ and Tripartite motif containing 17 (Trim17).^44^ Casitas B-lineage lymphoma (Cbl),^45^ and Itch^46^ work as an E3 ligase of c-FLIP. We also investigated the E3 ligase expression of c-FLIP and Mcl-1. However, combined treatment with carboplatin and thioridazine did not change E3 ligase expression of *β*-TrCP, Cbl, and Itch (Supplementary Figure S3). Further investigation is required to determine whether other E3 ligases or unknown E3 ligases might be involved in the degradation of c-FLIP and Mcl-1 expression.

Collectively, these results suggest that carboplatin plus thioridazine induces apoptosis in human head and neck cancer cells. The augmentation of proteasome activity by mitochondrial ROS-mediated PSMA5 expression induced downregulation of c-FLIP and Mcl-1 expression in carboplatin plus thioridazine-treated cells. Therefore, the combined modality approach with a low concentration of carboplatin and thioridazine may reduce the occurrence of unexpected side effects and improve the drug's anticancer effects.

## Materials and methods

### Cells and materials

Human head and neck cancer AMC-HN4 cells were obtained from Asan Medical Center. MDA-MB-231, U87MG, and EA.hy926 cells were purchased from the American Type Culture Collection (Manassas, VA, USA). Primary cultured human mesangial cells (Cryo NHMC) were purchased from Clonetics (San Diego, CA, USA). The cells were cultured in Dulbecco's modified Eagle's medium that contained 10% fetal bovine serum, 20 mM Hepes buffer, and 100 *μ*g/ml gentamicin. The PCR primers were purchased from Macrogen, Inc. (Seoul, Korea), and the other chemicals were purchased from Sigma (St. Louis, MO, USA). NAC and Trolox were obtained from Calbiochem (San Diego, CA, USA). The anti-Bcl-2, anti-Bcl-xL, anti-Mcl-1, anti-XIAP, anti-Nrf2, and anti-PARP antibodies were purchased from Santa Cruz Biotechnology (Santa Cruz, CA, USA). The anti-cleaved caspase-3 and anti-cIAP1 antibodies were obtained from Cell Signaling Technology (Beverly, MA, USA). The anti-pro-caspase-3 and anti-c-FLIP antibodies was obtained from ALEXIS Corporation (San Diego, CA, USA). The anti-PSMA5 antibody was purchased from Cell Signaling Technology. The anti-peroxiredoxin-SO_3_ antibody was purchased from AbFRONTIER (Seoul, Korea). The anti-actin antibody was obtained from Sigma. The human Mcl-1 and c-FLIP expression vectors were constructed as described previously.^47, 48^

### Flow cytometry analysis

For flow cytometry, the cells were resuspended in 100 *μ*l of phosphate-buffered saline (PBS), and 200 *μ*l of 95% ethanol was added while the cells were being vortexed. Then, the cells were incubated at 4 °C for 1 h, washed with PBS, resuspended in 250 *μ*l of 1.12% sodium citrate buffer (pH 8.4) with 12.5 *μ*g of RNase and incubated for an additional 30 min at 37 °C. The cellular DNA was then stained by adding 250 *μ*l of a propidium iodide solution (50 *μ*g/ml) to the cells for 30 min at room temperature. The stained cells were analyzed by fluorescent-activated cell sorting on a FACScan flow cytometer to determine the relative DNA content, which was based on the red fluorescence intensity. We provide the FACS plots in Supplementary Figure S4–S6.

### Western blot analysis

For the western blot experiments, the cells were washed with cold PBS and lysed on ice in modified RIPA buffer (50 mM Tris-HCl pH 7.4, 1% NP-40, 0.25% Na-deoxycholate, 150 mM NaCl, 1 mM Na_3_VO_4_, and 1 mM NaF) containing protease inhibitors (100 *μ*M phenylmethylsulfonyl fluoride, 10 *μ*g/ml leupeptin, 10 *μ*g/ml pepstatin, and 2 mM EDTA). The lysates were centrifuged at 10 000 × *g* for 10 min at 4 °C, and the supernatant fractions were collected. The proteins were separated by SDS-PAGE electrophoresis and transferred to Immobilon-P membranes. The specific proteins were detected using an enhanced chemiluminescence western blotting kit according to the manufacturer's instructions.

### Determination of the synergy and cell viability assay

The XTT assay was employed to measure cell viability using a WelCount Cell Viability Assay Kit (WelGENE, Daegu, Korea). In brief, the reagent was added to each well and was then measured with a multi-well plate reader (at 450 nm/690 nm).

### 4′,6′-Diamidino-2-phenylindole (DAPI) staining for nuclei condensation and fragmentation

To examine the cellular nuclei, the cells were fixed with 1% paraformaldehyde on glass slides for 30 min at room temperature. After fixation, the cells were washed with PBS and a 300 nM DAPI solution (Roche, Mannheim, Germany) was added to the fixed cells for 5 min. After the nuclei were stained, the cells were examined by fluorescence microscopy.

### The DNA fragmentation assay

A cell death detection ELISA plus kit (Boerhringer Mannheim; Indianapolis, IN, USA) was used to determine the level of apoptosis by detecting fragmented DNA within the nuclei of thioridazine-treated cells, carboplatin-treated cells, or cells that were treated with a combination of carboplatin and thioridazine. In brief, each culture plate was centrifuged for 10 min at 200 × *g*, the supernatant was removed, and the cell pellet was lysed for 30 min. Then, the plate was centrifuged again at 200 × *g* for 10 min and the supernatant, which contained the cytoplasmic histone-associated DNA fragments, was collected and incubated with an immobilized anti-histone antibody. The reaction products were incubated with a peroxidase substrate for 5 min and were measured by spectrophotometry at 405 and 490 nm (reference wavelength) with a microplate reader. The signals in the wells containing the substrate alone were subtracted as the background.

### Asp-Glu-Val-Asp-ase (DEVDase) activity assay

To evaluate the DEVDase activity, cell lysates were prepared after their respective treatments with carboplatin in the presence or absence of thioridazine. Assays were performed in 96-well microtiter plates by incubating 20 *μ*g of the cell lysates in 100 *μ*l of reaction buffer (1% NP-40, 20 mM Tris-HCl, pH 7.5, 137 mM NaCl, 10% glycerol) containing a caspase substrate (Asp-Glu-Val-Asp-chromophore-p-nitroanilide (DVAD-pNA)) at 5 *μ*M. The lysates were incubated at 37 °C for 2 h. Thereafter, the absorbance at 405 nm was measured with a spectrophotometer.

### Reverse transcription polymerase chain reaction (RT-PCR)

Total RNA was isolated using the TriZol reagent (Life Technologies; Gaithersburg, MD, USA), and the cDNA was prepared using M-MLV reverse transcriptase (Gibco-BRL; Gaithersburg, MD, USA) according to the manufacturers' instructions.^49, 50^ The following primers were used for the amplification of human c-FLIP, Mcl-1, PSMA5, and actin: c-FLIP (sense) 5′-CGGACTATAGAGTGCTGATGG-3′ and (antisense) 5′-GATTATCAGGCAGATTCCTAG-3′ Mcl-1 (sense) 5′-GCGACTGGCAAAGCTTGGCCTCAA-3′ and (antisense) 5′-GTTACAGCTTGGATCCCAACTGCA-3′ PSMA5 (sense) 5′-CTTGCAAGAAGTTTATCACAAGTCT-3′ and (antisense) 5′-GAAATTCTGGCCAGGCTGC-3′ and actin (sense) 5′-GGCATCGTCACCAACTGGGAC-3′, and (antisense) 5′-CGATTTCCCGCTCGGCCGTGG-3′. PCR amplification was carried out using the following cycling conditions: 94 °C for 3 min followed by 17 (actin) or 23 cycles (c-FLIP, and Mcl-1, and PSMA5) of 94 °C for 45 s; 58 °C for 45 s; 72 °C for 1 min; and a final extension at 72 °C for 10 min. The amplified products were separated by electrophoresis on a 1.5% agarose gel and detected under UV light.

### Proteasome activity assay

The chymotryptic proteasome activities were measured with Suc-LLVY-AMC (chymotryptic substrate, Biomol International, Plymouth Meeting, PA, USA). The cells were collected, washed with PBS and lysed. A mixture containing 1 *μ*g of protein from the cell lysate in 100 mM Tris-HCl (pH 8.0), 10 mM MgCl_2_, and 2 mM ATP was incubated at 37 °C for 30 min with 50 *μ*M Suc-LLVY-AMC. Enzyme activity was measured with a fluorometric plate reader at an excitation wavelength of 380 nm and an emission wavelength of 440 nm.

### DNA transfection and luciferase assay

Transient transfection was performed in six-well plates. One day before transfection, AMC-HN4 cells were plated at ~6080% confluence. The PSMA5/-277-luc and ARE-luc plasmids were transfected into cells using Lipofectamine 2000 (Invitrogen, Carlsbad, CA, USA). To assess promoter-driven expression of the luciferase gene, cells were collected and disrupted by sonication in lysis buffer (25 mM Tris-phosphate, pH 7.8, 2 mM EDTA, 1% Triton X-100, and 10% glycerol), and aliquots of the supernatant were used to analyze the luciferase activity according to the manufacturer's instructions (Promega, Madison, WI, USA).

### Preparation of cytosol and nuclear extracts

Following the required treatments, AMC-HN4 cells were trypsinized and suspended in buffer A (10 mM HEPES at pH 7.9, 10 mM KCl, 0.1 mM EDTA, 0.1 mM EGTA, 1 mM DTT, 0.5 mM PMSF). After incubation on ice for 30 min, the cells were centrifuged at 2500 rpm for 3 min to obtain a nuclear pellet. The supernatant fractions were collected as the cytosol extract. Buffer C (20 mM HEPES at pH 7.9, 0.4 M NaCl, 1 mM EDTA, 1 mM DTT, and 1 mM PMSF) was added, followed by a rotation for 30 min at 4 °C. The resulting lysates were centrifuged at 12 000 rpm at 4 °C for 5 min. The supernatant fractions were collected as the nuclear extract.

### Small interfering RNAs

The GFP (control), Nrf2, and PSMA5 small interfering RNA (siRNA) duplexes used in this study were purchased from Santa Cruz Biotechnology. The cells were transfected with siRNA using Oligofectamine Reagent (Invitrogen, Carlsbad, California, USA) according to the manufacturer's recommendations.

### Measurement of ROS

Intracellular accumulation of ROS was determined using the fluorescent probes 2′, 7′-dichlorodihydrofluorescein diacetate (H_2_DCFDA) and Mitosox Red. The AMC-HN4 cells were treated with carboplatin plus thioridazine, and then, the cells were stained with the H_2_DCFDA fluorescent dye or Mitosox Red for an additional 10 min, followed by trypsinization and resuspension in PBS. The fluorescence was measured at specific time intervals with a flow cytometer (Becton–Dickinson; Franklin Lakes, NJ, USA) or fluorescence microscope (Zeiss, NY, USA).

### Statistical analysis

The data were analyzed using one-way analysis of variance and *post hoc* comparisons (Student–Newman–Keuls) using the Statistical Package for Social Sciences 22.0 software (SPSS Inc.; Chicago, IL, USA).
