# Supplementary figures and images for: Thioridazine enhances sensitivity to carboplatin in human head and neck cancer cells through downregulation of c-FLIP and Mcl-1 expression
Source: Cell Death Dis. 2017 Feb 9;8(2):e2599–. doi: 10.1038/cddis.2017.8 (PMC5386499; doi:10.1038/cddis.2017.8)

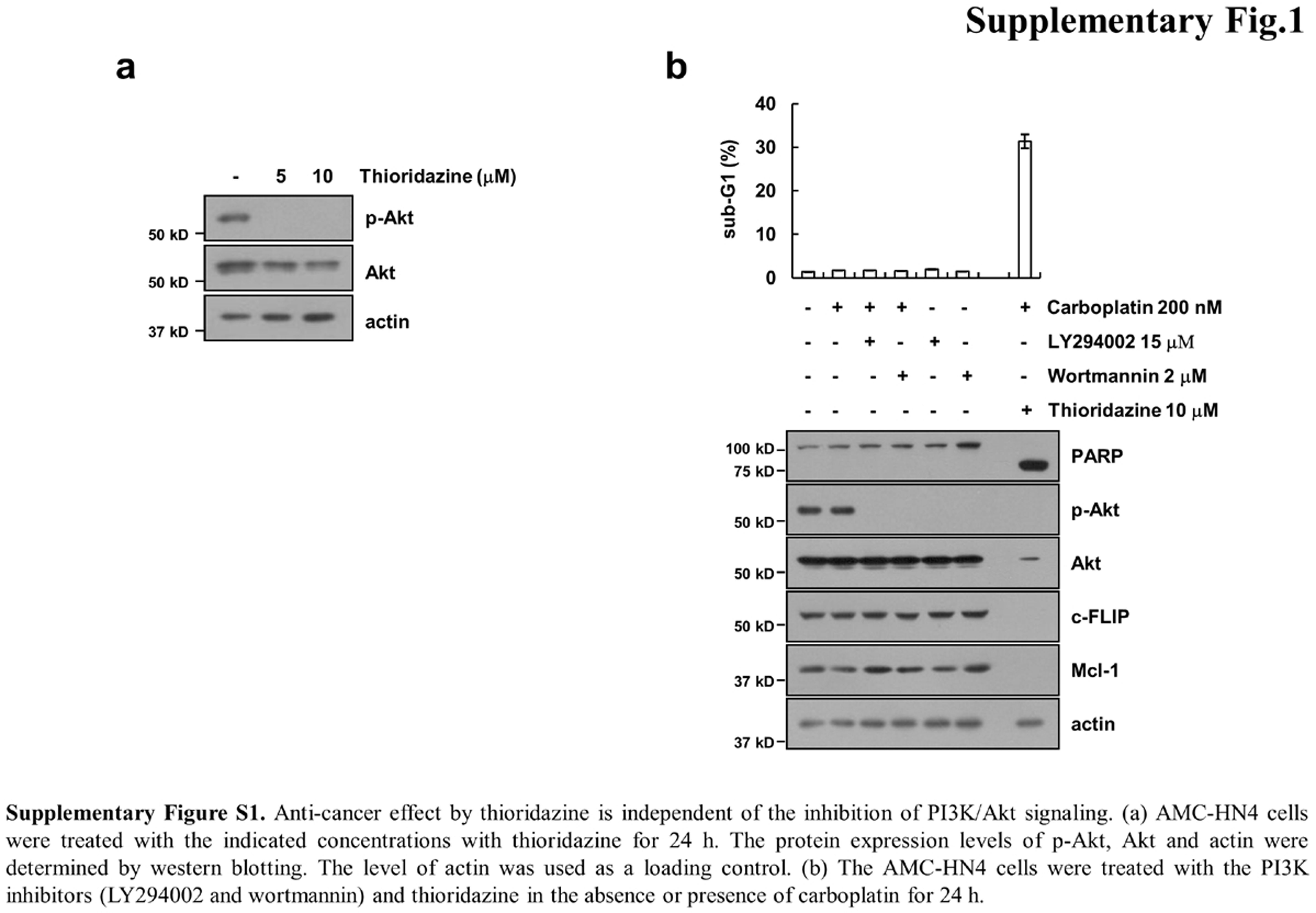

Supplement: Supplementary Figure S1 [file cddis20178x1.tif]

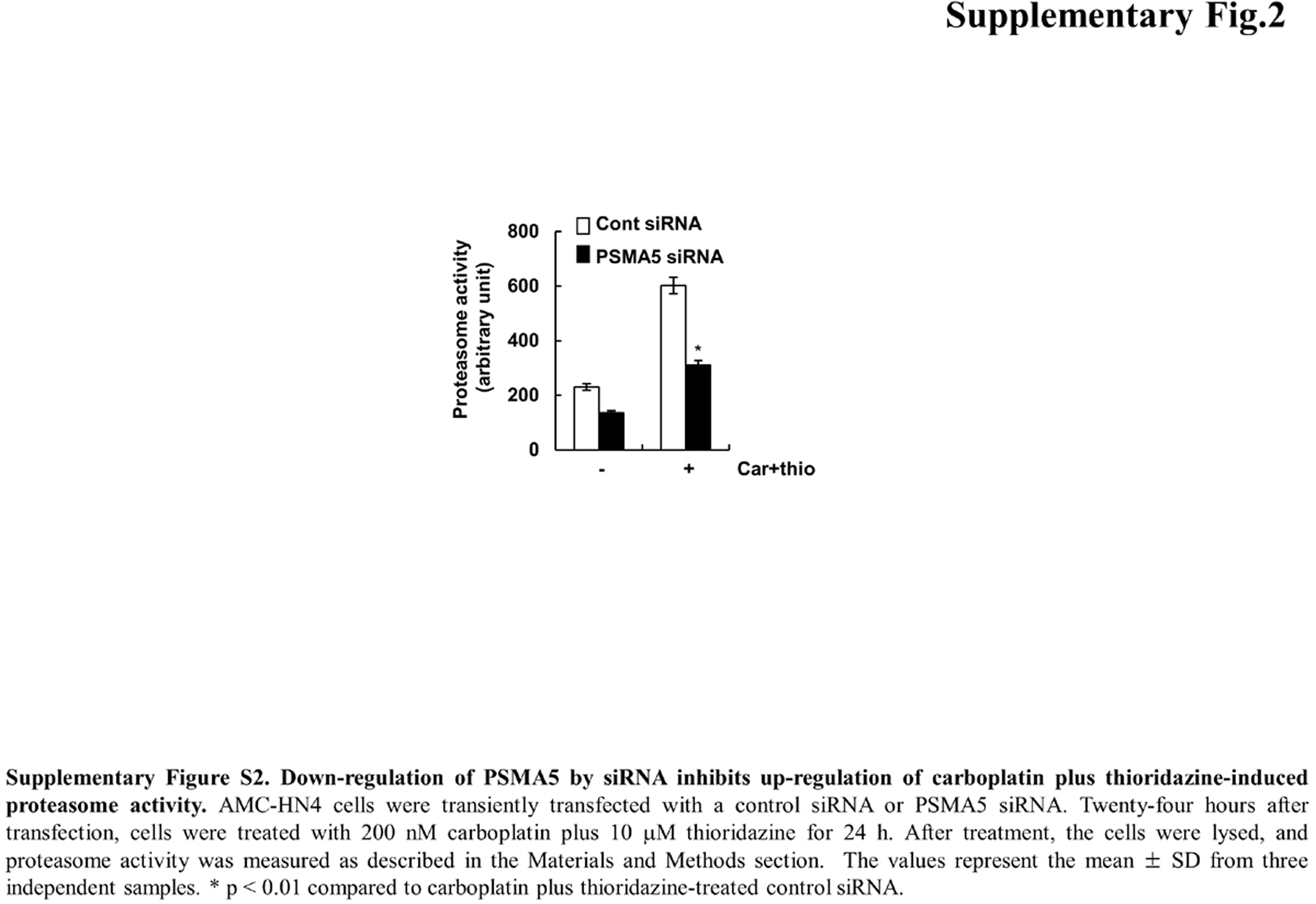

Supplement: Supplementary Figure S2 [file cddis20178x2.tif]

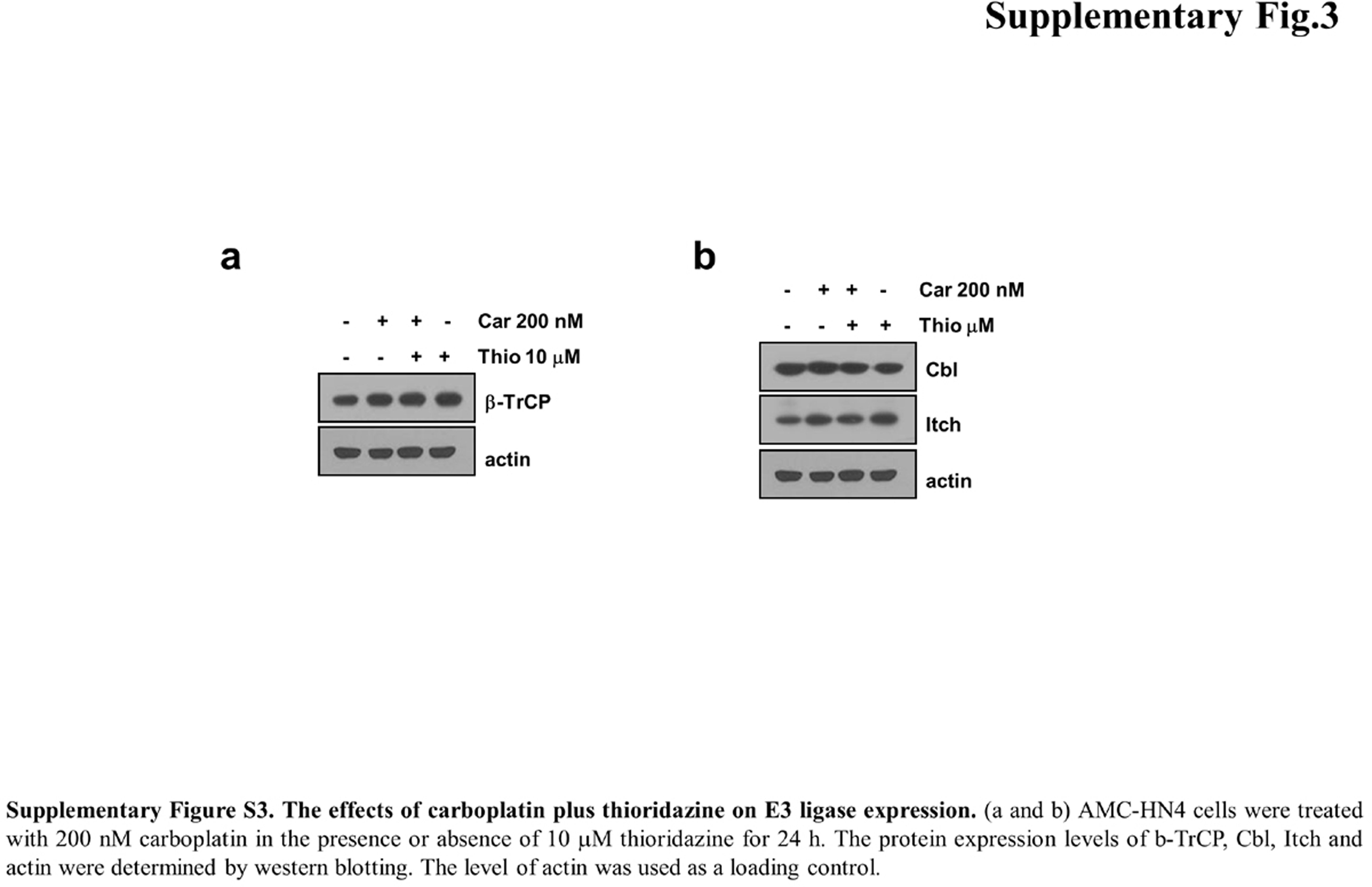

Supplement: Supplementary Figure S3 [file cddis20178x3.tif]

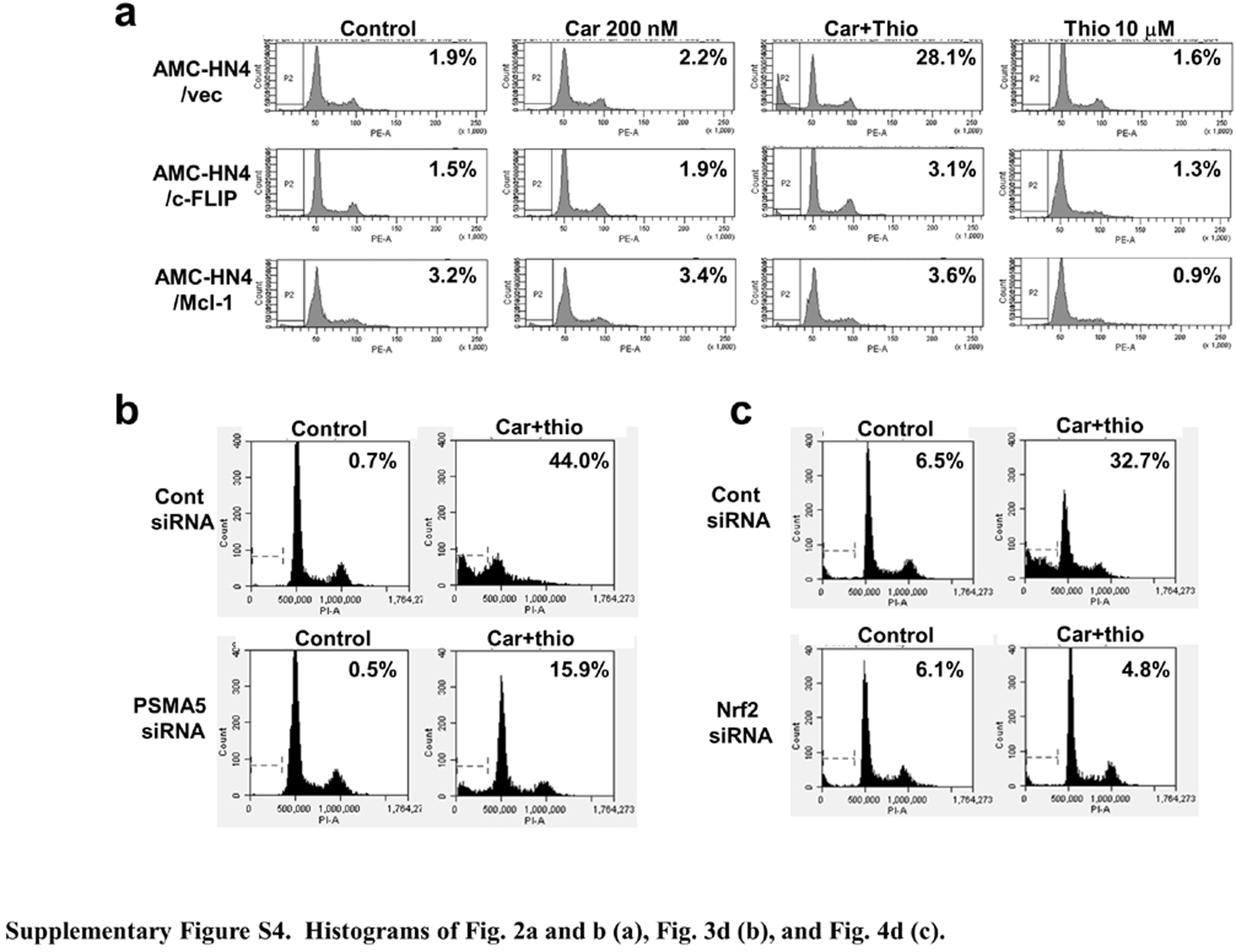

Supplement: Supplementary Figure S4 [file cddis20178x4.tif]

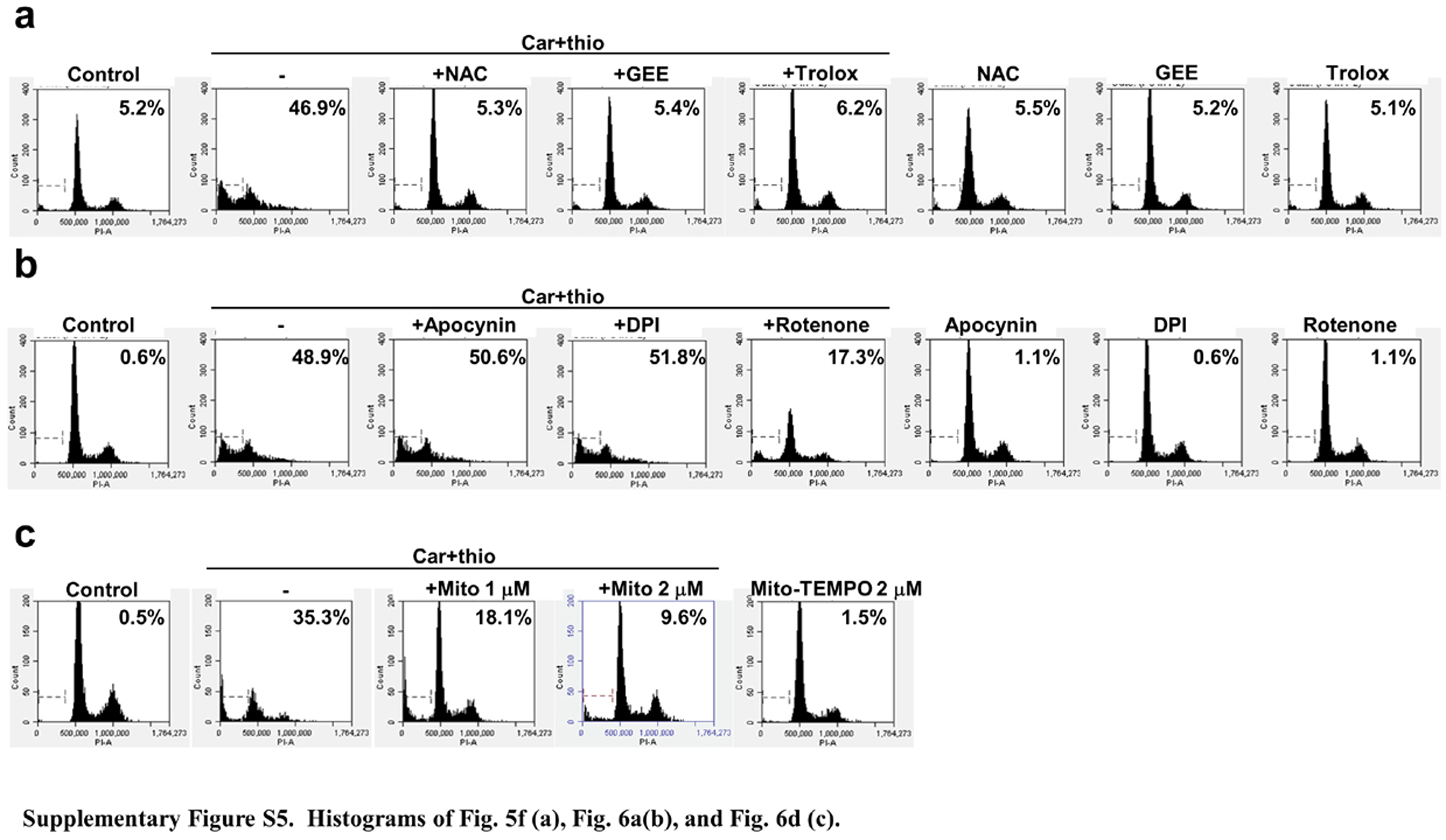

Supplement: Supplementary Figure S5 [file cddis20178x5.tif]

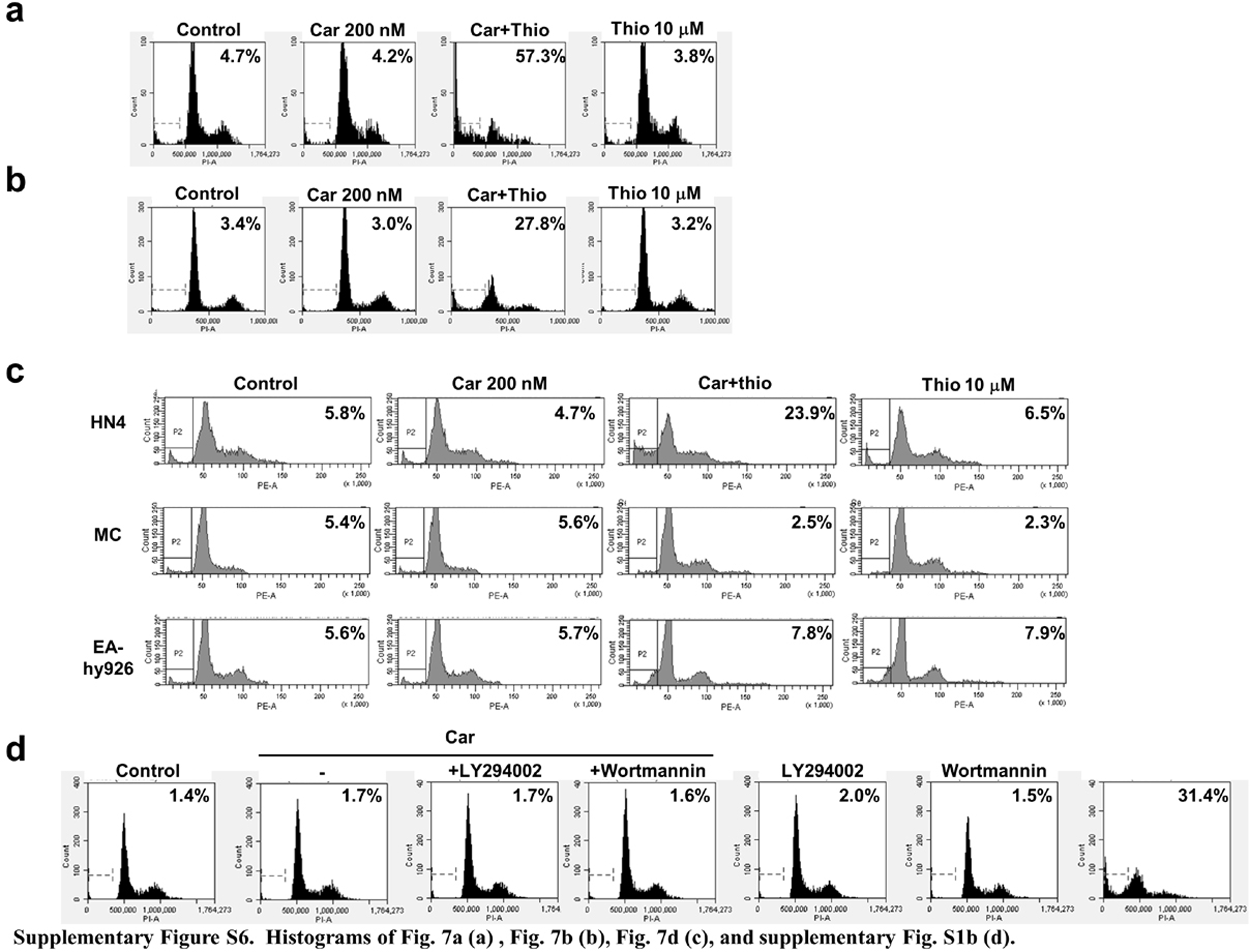

Supplement: Supplementary Figure S6 [file cddis20178x6.tif]
